# A Case of Pott’s Disease Complicated by Tuberculous Meningitis and Psoas Abscess Diagnosed by Computed Tomography-Guided Percutaneous Drainage

**DOI:** 10.7759/cureus.73750

**Published:** 2024-11-15

**Authors:** Ko Miyakoda, Ryohei Ono, Izumi Kitagawa

**Affiliations:** 1 Department of General Internal Medicine, Shonan Fujisawa Tokushukai Hospital, Fujisawa, JPN; 2 Department of Cardiovascular Medicine, Chiba University Graduate School of Medicine, Chiba, JPN

**Keywords:** computed tomography-guided percutaneous drainage, pott’s disease, psoas abscess, tuberculosis, tuberculous meningitis

## Abstract

Tuberculosis (TB) is an infectious disease caused by *Mycobacterium tuberculosis* (MTB). Disseminated TB can cause various types of complications. Extrapulmonary TB includes TB meningitis, abdominal TB, skeletal TB, Pott’s disease (spine), scrofula, and genitourinary TB. In particular, TB meningitis is a lethal complication of TB with a high mortality rate.

The patient was an 84-year-old Japanese man with a history of tuberculous pleurisy during childhood who presented with fever and altered mental status. Eight months prior, the patient was diagnosed with a urinary tract infection and treated with levofloxacin, but further investigation showed spondylitis. Although the causative microorganism was not identified as levofloxacin likely masked it, he was treated with linezolid and rifampicin for six weeks for the empiric diagnosis of bacterial pyogenic spondylitis; both medications are also antituberculous agents. At this visit, abdominal computed tomography (CT) revealed a left psoas abscess with calcification, and cerebral magnetic resonance imaging revealed multiple nodules with target signs. Cerebrospinal fluid (CSF) findings were consistent with TB meningitis, though the TB polymerase chain reaction, culture, and CSF cytology were all negative. He underwent CT-guided percutaneous drainage for the psoas abscess, and MTB was finally identified. Although he was treated with antituberculous agents including rifampicin, ethambutol, and isoniazid, the patient died seven months after admission as a result of the debilitating effects of tuberculous meningitis.

In our case, we should have tried to identify the causative organism in the diagnosis of pyogenic spondylitis. Especially in patients with a history of TB, the possibility of TB should always be considered. In addition, the use of antituberculous drugs may mask the presentation; therefore, biopsy or CT-guided drainage, as used in this case, should be considered for diagnostic accuracy.

## Introduction

Tuberculosis (TB) is an infectious disease caused by *Mycobacterium tuberculosis* (MTB) that can present with central nervous system (CNS) involvement such as meningitis, cerebral abscess, and tuberculoma [1]. Tuberculous meningitis is a lethal complication of MTB infection that accounts for approximately 5% of all extrapulmonary TB cases [2]. Tuberculous spondylitis, also known as Pott’s disease, is the most prevalent presentation among all forms of extrapulmonary TB [3]. While TB-associated primary psoas abscess is predominantly caused by hematogenous spread of MTB, secondary psoas abscess can result from MTB directly spread from Pott’s disease [4,5]. Computed tomography (CT)-guided percutaneous drainage of iliopsoas abscesses has been a useful method to accurately identify the causative pathogen, which, in turn, enables effective treatment of the infection [6]. In this report, we describe a complex case with Pott’s disease complicated by tuberculous meningitis and psoas abscess diagnosed by CT-guided percutaneous drainage.

## Case presentation

**Figure 1:**
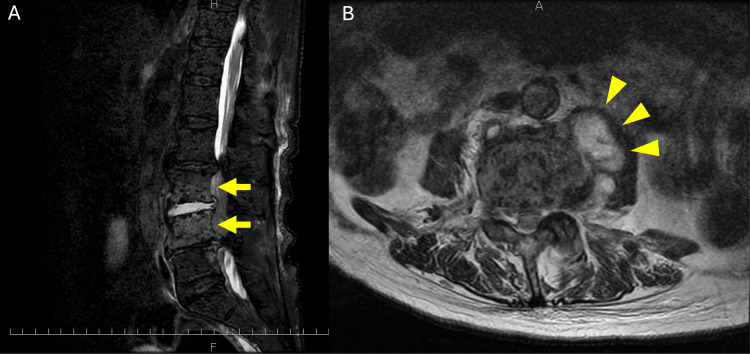
Lumbar magnetic resonance imaging scan. (A) T2-weighted iterative decomposition of water and fat with echo asymmetry and least-squares estimation imaging showing high signal at L3/4 (arrows). (B) T2-weighted fast recovery fast spin-echo revealing imaging paravertebral mass (arrows).

An 84-year-old Japanese man with a history of advanced prostate cancer and tuberculous pleurisy during childhood was admitted to our hospital with a fever and altered mental status. The patient was administered antituberculosis agents during childhood. His family history was unremarkable. Seven years before admission, the patient was diagnosed with prostate cancer. He initially underwent radiation therapy; however, bone scintigraphy revealed bone metastasis one year later. The patient was administered leuprorelin and enzalutamide. One year and three months before admission, chest CT revealed pulmonary metastases. Eight months prior, the patient had presented to another hospital with fever and back pain. The physician diagnosed him with a urinary tract infection and prescribed levofloxacin for seven days. However, the urine culture was negative for pathogens. The patient’s fever and back pain persisted, and oxycodone proved to be ineffective; therefore, he visited our hospital and was promptly admitted. Lumbar magnetic resonance imaging (MRI) revealed high signal intensity at L3/4 and paravertebral masses (Figure 1).

The patient was empirically diagnosed with bacterial pyogenic spondylitis, as blood cultures were negative and we considered that levofloxacin might mask the causative organism, and he was treated with linezolid and rifampicin for six weeks. After treatment, his symptoms subsided, and he was discharged. He was followed up monthly without antibiotics and had no symptoms; however, his serum C-reactive protein (CRP) level remained high between 1.13 mg/dL and 2.87 mg/dL (normal range = 0-0.30 mg/dL).

**Figure 2:**
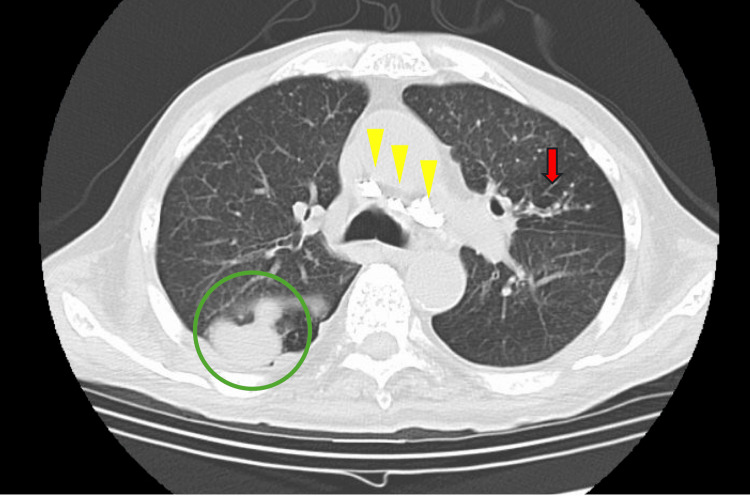
Chest computed tomography on admission. Chest computed tomography on admission showing mass lesions as pulmonary metastases in the right lung field (circled), calcified mediastinal lymph nodes (arrowheads), and a tree-in-bud appearance (arrow).

Ten days before this visit, the patient had gradually lost appetite and developed insomnia. He was referred to our hospital with a fever and altered mental status. On arrival, he was febrile (38.0℃) with a heart rate of 80 beats per minute, blood pressure of 116/56 mmHg, and oxygen saturation of 97% on room air. His Glasgow Coma Scale score was E2V3M4. Physical examination revealed neck stiffness and spine tenderness, but no jolt accentuation, Kernig’s sign, or psoas sign. Chest CT revealed mass lesions as pulmonary metastases in the right lung field, calcified mediastinal lymph nodes, and a tree-in-bud appearance (Figure 2).

**Figure 3:**
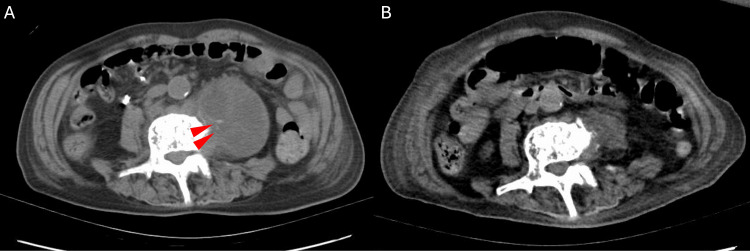
Abdominal computed tomography on admission and five months after the admission. (A) Abdominal computed tomography on admission revealing abscess with calcification (arrowheads) in the left psoas muscle. (B) Abdominal computed tomography five months after the admission showing abscess improvement.

Laboratory tests revealed the following: white blood cell count, 7,100/μL (normal range = 4,000-9,000/μL); CRP level, 3.96 mg/dL; and procalcitonin level, 0.026 ng/mL (normal range = <0.05 ng/mL). Urinalysis revealed no evidence of pyuria. Head CT revealed no abnormalities, including tumors or bleeding; however, abdominal CT revealed a left psoas abscess with calcification (Figure 3, Panel A).

**Figure 4:**
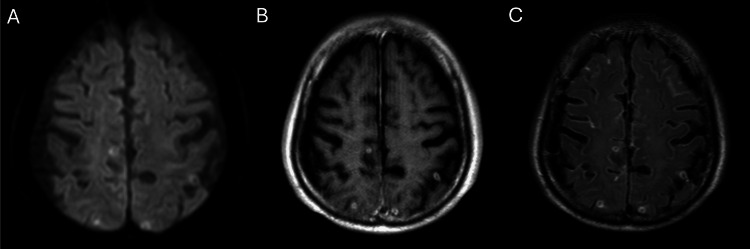
Cerebral magnetic resonance imaging. Cerebral magnetic resonance imaging demonstrating multiple nodules with ring-like enhancement (target sign). (A) Diffusion-weighted imaging. (B) Gadolinium-enhanced T1-weighted imaging. (C) Contrast-enhanced fat-saturated fluid-attenuated inversion recovery imaging.

Cerebral MRI revealed multiple nodules with ring-like enhancements (target signs) (Figure 4).

The cerebral MRI findings led us to suspect tuberculous meningitis. Subsequently, cerebrospinal fluid (CSF) evaluation revealed an increased cell count of 60/μL (normal range = 0-15/μL) with 55% mononuclear cells, protein concentration of 161.2 mg/dL (normal range = 15-40 mg/dL), adenosine deaminase of 13 U/L (normal range = 8-16 U/L), and glucose level of 39 mg/dL (normal range = 50-70 mg/dL) with an opening pressure of 50 mmH_2_O. The paired serum glucose level was 125 mg/dL. The TB polymerase chain reaction, culture, and CSF cytology were all negative.

Based on his medical history of TB pleurisy, cerebral MRI, and CSF findings, tuberculous meningitis was strongly suspected. The patient was then admitted for further evaluation and treatment. Despite the administration of rifampicin, ethambutol, isoniazid, and dexamethasone, the patient remained unconscious. Repeated CSF tests were performed a total of six times every two weeks and the results showed a decreased cell count to 33/μL and increased glucose levels (53-61 mg/dL). Repeated evaluations of acid-fast bacterial cultures of CSF, sputum, and blood were negative.

On day 10, he underwent CT-guided percutaneous drainage for the psoas abscess, and MTB was identified by Ziehl-Neelsen staining with Gaffky 5. Antibiotic susceptibility testing for MTB was performed and all antituberculosis drugs including rifampicin, ethambutol, isoniazid, and levofloxacin were susceptible. Although the abscess had shrunk after drainage, his CRP level remained positive and unconsciousness persisted. His physical activity gradually declined, and he became almost bedridden. Repeat head MRI three months after admission showed the disappearance of scattered ring-enhancing nodules in the brain surface region compared with the previous scan, but granulomas had formed in the arachnoid cavity, with atrophy of the right temporal lobe and high signal on T2-weighted image. Atrophy of the entire brain parenchyma was also observed, suggesting the effects of inflammation from extensive tuberculous meningitis. Reexamination of the abdominal CT scan after five months showed improvement in the abscess (Figure 3, Panel B). He received seven months of antituberculosis treatment but died seven months after admission as a result of the debilitating effects of tuberculous meningitis.

## Discussion

Here, we report a case of Pott’s disease complicated by tuberculous meningitis and tuberculous psoas abscess. Based on rigorous reviews, we identified a case of these three conditions concomitantly, but tuberculous meningitis occurred one month after surgery for spinal TB and extensive psoas abscesses [7]. We could not find epidemiological data on the prevalence of these three conditions together.

Tuberculous meningitis has a high mortality rate of approximately 30%, even with appropriate antituberculous treatment [8]. Pott’s disease is the most common form of extrapulmonary TB and may involve paravertebral extension with the formation of a paravertebral abscess. Differential diagnoses for Pott’s disease include pyogenic and fungal infections, sarcoidosis, metastasis, and lymphoma [9].

Regarding treatment, the Japanese Tuberculosis Treatment Guideline recommends nine months of isoniazid, rifampicin plus ethambutol, or streptomycin instead of pyrazinamide if the patient is over 80 years old because of the risk of liver damage. Moreover, according to the American guidelines, some experts avoid the use of pyrazinamide during the intensive phase among patients >75 years of age. In such cases, the initial regimen consists of isoniazid, rifampicin, and ethambutol. If pyrazinamide is not used during the intensive phase, then the total duration of TB treatment should be extended to at least nine months [10]. Therefore, the patient was treated with isoniazid, rifampicin, and ethambutol.

Our case was very complicated because the patient was first empirically diagnosed with bacterial pyogenic spondylitis, considering the possibility of masking the microorganism due to previous use of levofloxacin, and treated empirically with linezolid and rifampicin. Of note, these antibiotics were all antituberculous agents, and he was partially treated and later complicated by tuberculous meningitis. Furthermore, repeated cultures of CSF were negative in our case, but another previous study highlighted the crucial role of repetitive sample collection including CSF culture or polymerase chain reaction and biopsy for other tissues to improve diagnostic sensitivity [11]. If cultures remain negative but the clinician suspects tuberculous meningitis, a consensus tuberculous meningitis diagnosis is useful [12]. The criteria include the clinical entry criteria plus other diagnostic scores such as CSF criteria, cerebral imaging criteria, and evidence of tuberculosis elsewhere. Cerebral imaging criteria include hydrocephalus, basal meningeal enhancement, tuberculoma, infarct, and pre-contrast basal hyperdensity. Based on the total diagnostic score, tuberculous meningitis can be diagnosed even if the CSF culture is negative.

In addition, our case is very educational because the patient was initially difficult to diagnose but had several typical tuberculous features characterized by multiple modalities. First, the head MRI showed a “target sign” characterized by tuberculomas. “Target sign” is central enhancement accompanied by peripheral rim enhancement on post-contrast MRI [13]. Although the imaging features of a CNS tuberculoma depend on the stage of the lesion, the “target sign,” consisting of a ring-enhancing lesion with an additional central area of enhancement or calcification, has been described as characteristic of tuberculomas [14,15]. Other differential diagnoses for tuberculoma include neurocysticercosis, glioma, CNS lymphoma, and metastatic brain tumors [16]. In our case, the patient had no history of parasitic infection, which was not consistent with neurocysticercosis, and glioma was also unlikely because it is often associated with a single tumor. CNS lymphoma and metastatic brain tumors cannot be excluded as we did not perform a biopsy, but tuberculoma was most likely based on the identified microorganism. Second, calcification of paraspinal masses is highly suggestive of TB [3,17]. Our patient had calcification in the psoas muscle, which was the key finding for a suspected TB infection. Based on these findings, we suspected a TB infection; however, blood, sputum, and CSF cultures were initially negative. This is probably because the patient had previously used antituberculous antibiotics such as levofloxacin and linezolid, which may have been partially effective and masked the infection. CT-guided percutaneous drainage of iliopsoas abscesses is a relatively simple and safe way to identify the causative microorganism, including TB, and we performed it for both diagnosis and treatment and finally identified TB infection [17].

## Conclusions

We reported a case of Pott’s disease complicated by tuberculous meningitis and psoas abscess. Especially in patients with a history of TB, the possibility of TB should always be considered. Typical imaging features such as target signs (central enhancement with peripheral rim enhancement on post-contrast MRI) or calcifications in the psoas muscle help to accurately diagnose TB infection. In addition, the use of antituberculous drugs may mask the presentation; therefore, biopsy or CT-guided drainage, as used in this case, should be considered for the diagnosis. Our reflections have also highlighted the importance of early diagnosis and multidisciplinary management of complex cases of TB, which can lead to fatal outcomes.
